# 

**Published:** 1862-02

**Authors:** 


### CHICAGO MEDICAL JOURNAL.

**Terms, $2.00 per Annum.**

### CONTENTS OF THE FEBRUARY NUMBER.

SELECTED.

PAGE.

Food—Sleep—Profuse Perspiration. From Dr. Ware's Lecture on Therapeutics.......... 61

Homœopathy in Military Hospitals.......... 71

Medicine among the Mormons and the Indians of North America........ 77

On the Eliminination of Mercury from the System.......... 85

Notes of cases in Surgery. By Frederic C. Skey, Esq., F. R. S., Surgeon to St. Bartholomew's Hospital.......... 86

ORIGINAL COMMUNICATIONS.

Valedictory to the Graduates of Rush Medical College—Feb. 5th, 1862. By J. W. Freer, M. D., Prof. of Physiology and Surgical Anatomy 101

De Witt County Medical Society.......... 118

EDITORIAL.

Editorial and Miscellaneous.......... 119

Graduates of Rush Medical College—Session of 1861–62.......... 121

